# Charting the Proteins of Oropouche Virus

**DOI:** 10.3390/v17111434

**Published:** 2025-10-28

**Authors:** Sunil Thomas

**Affiliations:** Lankenau Institute for Medical Research, Wynnewood, PA 19096, USA; thomass-02@mlhs.org or suntom2@gmail.com

**Keywords:** Oropouche virus (OROV), Oropouche virus disease, sloth fever, protein mapping, antigenic proteins, structural biology

## Abstract

Oropouche virus (OROV) is an emerging arbovirus responsible for Oropouche fever, also known as sloth fever, a febrile illness that can lead to recurrent outbreaks in affected regions. Endemic to parts of South and Central America, OROV is primarily transmitted by biting midges (*Culicoides paraensis*), although mounting evidence implicates mosquitoes, particularly the Culex and Aedes species, as additional vectors. Recent ecological disturbances—such as deforestation, urbanization, and climate change—have driven significant shifts in vector population dynamics, contributing to the expanded geographic range and increased transmission of OROV. Notably, recent reports of OROV infections among American and European travelers to Cuba highlight the virus’s growing potential for international dissemination and underscore its significance as a global health concern. OROV is an enveloped orthobunyavirus within the Peribunyaviridae family, possessing a tripartite, single-stranded, negative-sense RNA genome composed of the S (small), M (medium), and L (large) segments. These segments encode the nucleocapsid (N) protein, glycoproteins (Gn and Gc), and RNA-dependent RNA polymerase, respectively. Despite increasing incidence and potential for global spread, no licensed vaccines or antiviral therapies currently exist, and effective diagnostic tools remain limited. Furthermore, although human-to-human transmission has not been observed, the absence of robust surveillance systems complicates timely outbreak detection and response. In this study, we present a comprehensive molecular characterization of OROV’s major structural proteins, with an emphasis on structural modeling and epitope prediction. By integrating bioinformatics approaches with available structural data, we identify key antigenic regions that could serve as targets for the development of serological diagnostics and vaccine candidates. Our findings contribute critical insights into the molecular virology of OROV and provide a foundational framework for future efforts aimed at the prevention, diagnosis, and control of this neglected tropical pathogen. These advancements are essential for mitigating the impact of OROV in endemic regions and reducing the risk of global emergence.

## 1. Introduction

Oropouche virus disease is a mosquito- and midge-borne arboviral illness caused by the Oropouche virus (OROV), a segmented, single-stranded, negative-sense RNA virus belonging to the genus Orthobunyavirus within the Peribunyaviridae family. The virus was first isolated in 1955 from a febrile human patient in the town of Oropouche, located near the Oropouche River and Oropouche Swamp in the southwestern region of Trinidad, Trinidad and Tobago [1,2]. Since its initial discovery, OROV has been increasingly recognized as a significant emerging pathogen across tropical and subtropical regions of the Western Hemisphere. The virus has been identified in several countries in South and Central America as well as parts of the Caribbean [3,4]. As of 2025, OROV disease has been documented in Argentina, Barbados, Bolivia, Brazil, Colombia, Cuba, Dominica, Ecuador, French Guiana, Guatemala, Guyana, Haiti, Panama, Peru, Trinidad and Tobago, and Venezuela [5]. In 2024, the CDC reported 21 travel-associated cases of Oropouche virus disease in U.S. citizens returning from Cuba, suggesting that the virus may be expanding its range and becoming increasingly relevant to international public health surveillance [6]. The Pan American Health Organization (PAHO) reported 12,786 cases of OROV infection across 11 countries in South America in 2025 alone, reflecting a substantial increase in case detection and possibly a broader geographic spread of the virus [7]. Given the rising incidence and the potential for further expansion into non-endemic regions, OROV represents a growing concern for regional and global health authorities, especially in the context of climate change, urbanization, and increasing international travel.

Oropouche fever is characterized by an acute febrile illness in humans, presenting with symptoms such as fever, headache, general malaise, muscle pain (myalgia), joint pain (arthralgia), sensitivity to light (photophobia), nausea, vomiting, and dizziness [8,9]. In some cases, it can lead to more severe conditions like encephalitis and meningitis [9,10]. The symptoms typically last from 2 to 7 days, though some patients may experience a recurrence [11]. These symptoms can be particularly debilitating, leading to considerable discomfort and disruption of daily activities. While the majority of individuals with Oropouche fever recover fully without long-term effects, the absence of a specific antiviral treatment or a vaccine means that management of the illness largely focuses on alleviating symptoms and providing supportive care [12,13].

The Oropouche virus has a sylvatic (wild) transmission cycle, primarily involving pale-throated sloths, non-human primates, and birds as reservoirs [14,15]. Transmission to humans is mainly mediated by midges of the species *Culicoides paraensis* [16,17], and possibly mosquitoes [12,18]. Despite this sylvatic cycle, the virus has been known to infect people in urban areas far from forested environments, also suggesting the presence of an urban transmission cycle. As of now, there is no documented evidence of human-to-human transmission of OROV [13].

The OROV is an enveloped virus with a tripartite genome, consisting of three single-stranded, negative-sense RNA segments. The small (S) segment encodes two overlapping open reading frames (ORFs), which produce the nucleocapsid protein (N) and the nonstructural (NS) protein NSs. NSs functions as a type I interferon inhibitor, helping the virus evade the host’s immune response [19]. The nucleoprotein is a significant structural protein that can be the target of diagnostics and therapeutics. The medium (M) segment encodes a polyprotein that is cleaved into structural glycoproteins Gn and Gc, as well as the NSm protein, whose role remains unclear. Gn and Gc form heterodimers and multimerize on the surface of the virion [20]. The Gc protein is a 939-amino-acid class II membrane fusion protein with 3–4 potential N-linked glycosylation sites, while Gn is a 290-amino-acid protein with one potential N-linked glycosylation site. Despite their importance, there is relatively little structural information available on Gc and Gn proteins in members of the Orthobunyavirus genus [2].

The lack of available vaccines makes it challenging to prevent OROV infections. This paper provides a comprehensive analysis of the protein structure of OROV, detailing the spatial arrangement of its constituent proteins. In addition to mapping the overall protein architecture, the study identified and characterized both B-cell and T-cell epitopes within the viral proteins. These epitopes are crucial as they are recognized by the immune system and play a significant role in the development of immune responses. Understanding the precise structure and location of these epitopes allows for more targeted approaches in the creation of diagnostics and vaccines. By leveraging this detailed protein mapping, researchers can design more effective diagnostic tools to detect OROV infections and develop vaccines that elicit a robust immune response, providing enhanced protection against this virus. This structural information is therefore pivotal for advancing both the prevention and management of OROV-related diseases.

## 2. Materials and Methods

### 2.1. Protein Sequence of Oropouche Virus

The sequences of the OROV were downloaded from the NCBI (https://www.ncbi.nlm.nih.gov/protein/, accessed on 8 August 2024) [21] protein database. The OROV proteins, accession numbers, and functions are shown in Table 1.

Conserved protein domains within the OROV were identified using the NCBI’s Conserved Domain Database (CDD) search tool, accessible at https://www.ncbi.nlm.nih.gov/Structure/cdd/wrpsb.cgi (accessed on 12 August 2024) [22]. The post-translational modification (PTM) of the proteins was determined using via prompt-based fine-tuning of a GPT-2 model (https://nsclbio.jbnu.ac.kr/tools/ptmgpt2, accessed on 7 October 2025) [23,24].

### 2.2. Protein Modeling

A comprehensive understanding of biological systems depends on grasping how protein complexes and networks operate, which requires a detailed examination of protein interactions and their quaternary structure. Protein plots, or snake diagrams, offer a two-dimensional view of a protein sequence, highlighting features like secondary structure [25]. To create these diagrams, we used Protter (http://wlab.ethz.ch/protter, accessed on 19 August 2024) [26], an interactive web-based tool. Protter allows users to integrate and visualize annotated and predicted protein sequence features, experimental proteomic data, and post-translational modifications onto the protein’s transmembrane topology. Users can select from various annotation sources, upload their own proteomics data files, choose peptides for targeted quantitative proteomics, and generate high-quality visualizations [27].

Phobius was used for the prediction of transmembrane topology and signal peptides from the amino acid sequence of proteins (https://phobius.sbc.su.se/, accessed on 13 August 2024) [28,29,30].

For the three-dimensional homology modeling, we employed AlphaFold ver.2 (https://colab.research.google.com/github/sokrypton/ColabFold/blob/main/AlphaFold2.ipynb, accessed on 12 August 2024) [31,32] and Phyre2 (version 2.0) [33] (http://www.sbg.bio.ic.ac.uk/phyre2, accessed on 15 August 2024) with default settings and visualized by RasMol version 2.7 [32]. The protein sequences of OROV were entered in FASTA format.

In addition to AlphaFold Ver. 2, for the three-dimensional homology modeling, we employed the iterative threading assembly refinement (I-TASSER) (https://zhanglab.ccmb.med.umich.edu/I-TASSER/, accessed on 12 November 2024) [34] with default settings. The protein sequences of OROV were entered in FASTA format. We determined the confidence score (C-score), template modeling score (TM-score), Root Mean Square Deviation (RMSD), and normalized B-factor using I-TASSER.

The normalized B-factor for a target protein is derived by applying a z-score normalization to the raw B-factor values. The B-factor itself reflects the degree of the inherent thermal mobility of residues or atoms in a protein. The normalized B-factor, referred to as the B-factor profile (BFP), is predicted through a combination of template-based assignment and profile-based prediction methods. According to the distribution and predictions of the BFP, residues with BFP values greater than 0 are considered less stable in experimental structures [35].

The C-score is a confidence metric used to assess the quality of models predicted by I-TASSER and is calculated based on the significance of threading template alignments and the convergence parameters from the structure assembly simulations. The C-score typically ranges from [−5, 2], with higher values indicating a model with greater confidence, and lower values suggesting lower confidence [36].

TM-score and RMSD are widely recognized metrics for assessing the structural similarity between two structures, commonly used to evaluate the accuracy of structure models when the native structure is available. When the native structure is unknown, however, it becomes essential to predict the quality of the model based on other criteria [37,38].

The B-cell epitope and T-cell epitope (MHC-I allele: HLA B35:01, MHC-II allele: HLA-DRB1; HLA DQA1*01:01/DQB1*02:01) was predicted using the IEDB Analysis Resource (http://tools.iedb.org, accessed on 5 September 2024) [39]. The BepiPred-2.0 tool was used for predicting B-cell epitopes from antigen sequences. BepiPred-2.0 is based on a random forest algorithm trained on epitopes annotated from antibody-antigen protein structures [40]. The T-cell epitope prediction tool available on the IEDB Analysis Resource uses various methods to predict MHC Class I and II epitopes, including a combined consensus approach that leverages strengths from individual methods like NN-align, SMM-align, and Combinatorial Library predictions to enhance accuracy. The B-cell epitopes are underlined; the T-cell epitopes are shaded in yellow.

The predicted solvent accessibility was determined using NetSurfP-3.0 (https://services.healthtech.dtu.dk/services/NetSurfP-3.0/, accessed on 22 October 2024) [41,42]. NetSurfP-3.0 predicts the secondary structure, solvent accessibility, disorder, and backbone geometry of any given protein based on its primary amino acid sequence. Using advanced protein language models and deep learning techniques, it provides accurate and rapid predictions of protein structural features. The resulting plot illustrates the protein’s backbone elements—helix, strand, and coil—along with regions of disorder and areas distinguished by hydrophobic or hydrophilic characteristics [42].

The OROV proteins underwent a comparative analysis with Madre de Dios virus and Schmallenberg virus proteins through the UniProt platform (https://uniprot.org/align, accessed on 11 September 2025) [43].

## 3. Results

### 3.1. Identification of Conserved Domains in Oropouche Virus Proteins

Protein sequences corresponding to the virus’s genomic segments were retrieved and analyzed using the NCBI’s Conserved Domain Database (CDD) platform to determine the presence of conserved functional and structural domains.

OROV is an enveloped, negative-sense RNA virus. Its genome is tri-segmented, consisting of three single-stranded RNA (ssRNA) molecules: large (L), medium (M), and small (S). Each segment encodes proteins essential for viral replication and assembly:

L segment: Encodes the RNA-dependent RNA polymerase, a key enzyme responsible for viral RNA transcription and replication.

M segment: Encodes two envelope glycoproteins, Gn and Gc, which are involved in viral attachment and entry into host cells. This segment also encodes a non-structural protein (NSm), believed to play a role in viral assembly or budding.

S segment: Encodes the nucleocapsid protein (N), which encapsidates the viral RNA, forming ribonucleoprotein (RNP) complexes essential for genome protection and packaging. This segment also encodes a non-structural protein, NSs.

The domain analysis helps to better understand the functional organization of the viral proteome and may aid in the identification of potential targets for antiviral development or vaccine design.

### 3.2. Gn Glycoprotein

**Figure 1:**
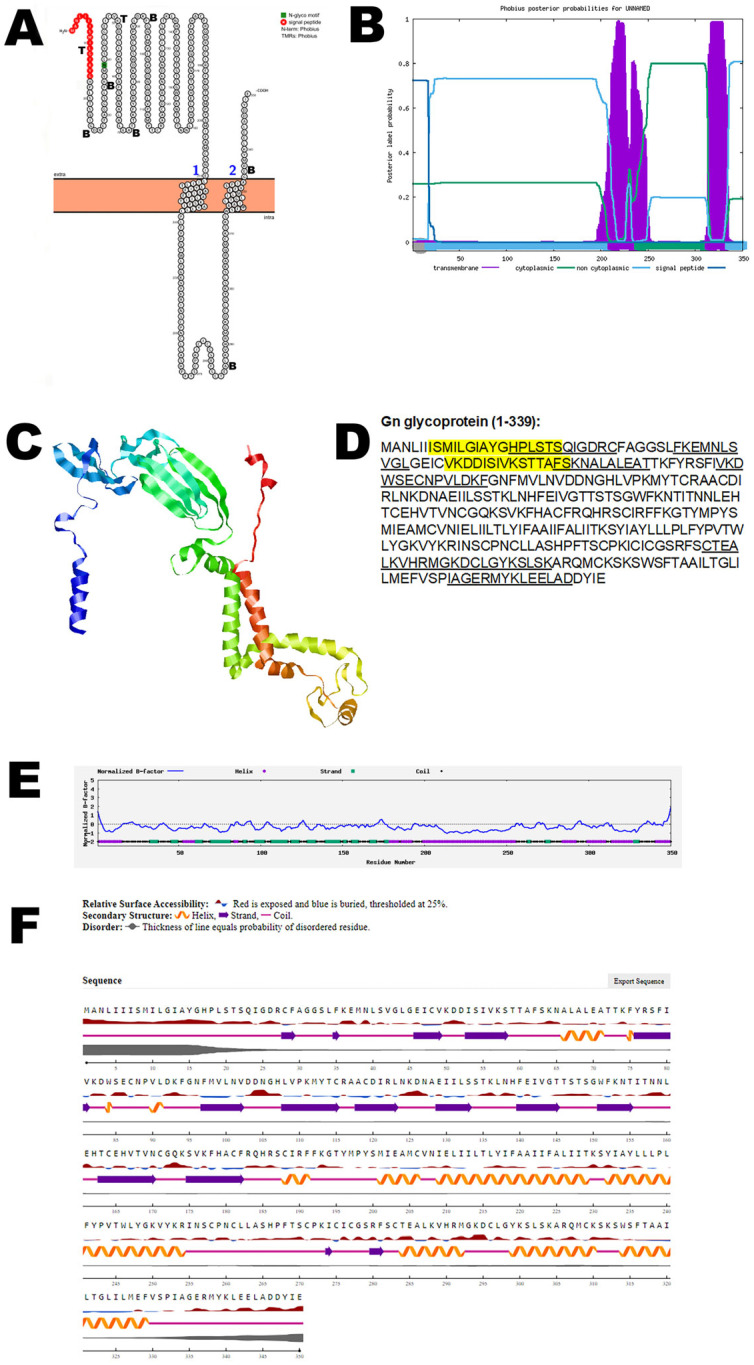
**The predicted structure of Gn glycoprotein of OROV.** (**A**) The topology of the OROV Gn glycoprotein as determined by Protter. The T-cell (T) and B-cell (B) epitope positions are marked in the figure. (**B**) The topology of OROV Gn glycoprotein as determined by Phobius. (**C**) The predicted protein structure of Gn glycoprotein (ribbon diagram) determined using the software AlfaFold (Version 2) (**D**) The B-cell (underlined) and T-cell epitope (yellow shaded) (Score > 0.3) of OROV Gn glycoprotein (Accession UYI36405; Amino acid: 1–350). (**E**) Normalized B-factor of Gn glycoprotein determined using I-TASSER. (**F**) The predicted solvent accessibility of Gn glycoprotein determined using NetSurfP-3.0.

The medium (M) segment of the RNA of OROV encodes a polyprotein that is cleaved into structural glycoproteins Gn and Gc, as well as the NSm protein. Gn glycoprotein is a structural protein of OROV and is the smallest glycoprotein. It is one of the envelope glycoproteins responsible for facilitating viral entry into host cells as well as release from host cells. Bioinformatic analysis using Protter demonstrated that the Gn glycoprotein has two transmembrane domains; the structure was confirmed using Phobius (Figure 1A,B). AlphaFold 2 predicted the 3D structure of the Gn glycoprotein (Figure 1C). The B-cell and T-cell epitopes are depicted in Figure 1D and Figure S1. The normalized B-factor of the Gn glycoprotein is shown in Figure 1E. The predicted solvent accessibility of Gn glycoprotein is plotted in Figure 1F. Some of the B-cell and T-cell epitopes overlap and may be considered vaccine candidates. The C-score, TM-score, and RMSD of Gn glycoprotein are shown in Table 2. The post-translational modification (PTM) of the protein is shown in Figure S7.

### 3.3. Gc Glycoprotein

**Figure 2:**
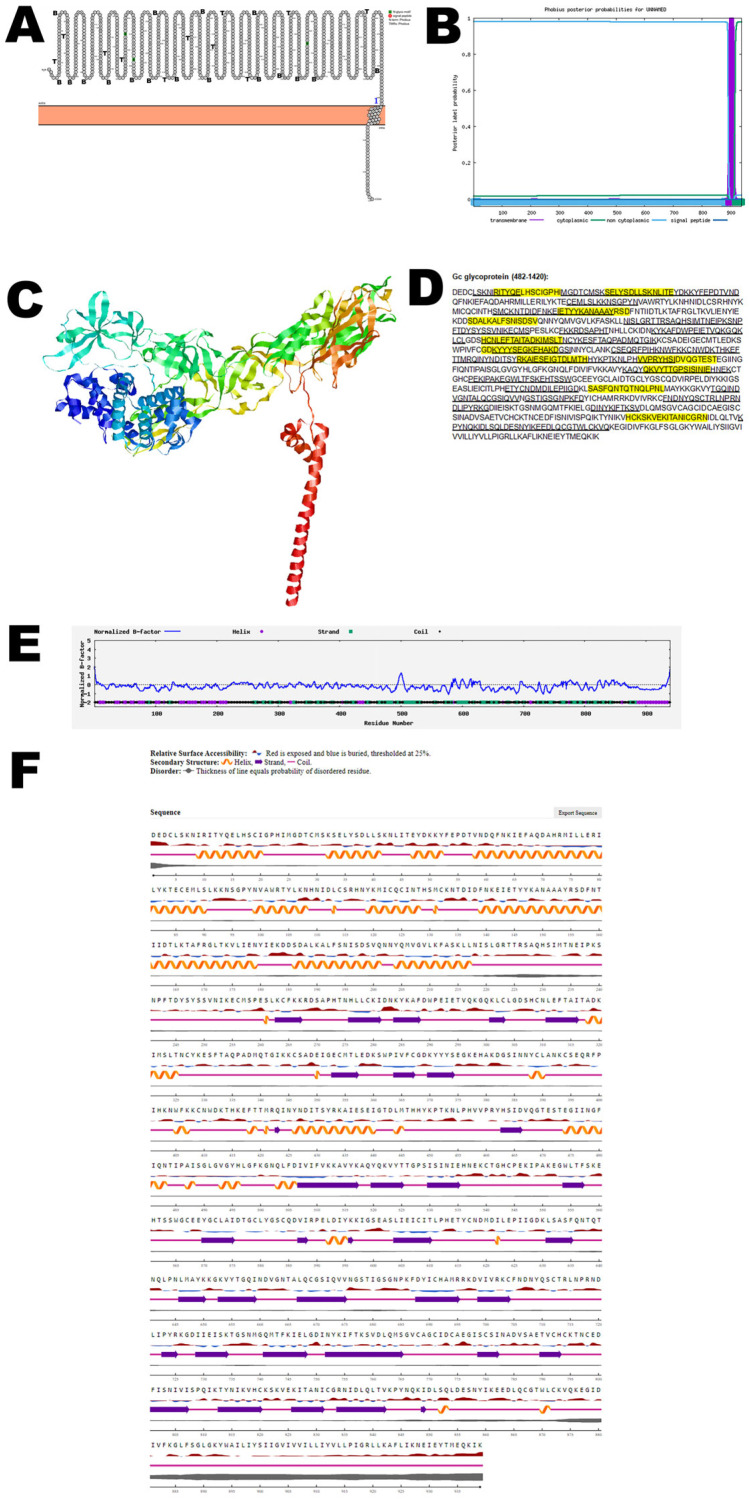
**The computationally predicted three-dimensional structure of the Gc glycoprotein of OROV.** (**A**) The topology of OROV Gc glycoprotein as determined by Protter. The T-cell (T) and B-cell (B) epitope positions are marked in the figure. (**B**) The topology of the OROV Gc glycoprotein as determined by Phobius. (**C**) The predicted protein structure of the Gc glycoprotein (ribbon diagram) determined using the software AlfaFold 2. (**D**) The B-cell (underlined) and T-cell epitope (yellow shaded) (Score > 0.5) of the OROV Gc glycoprotein (Accession UYI36405; Amino acid: 482–1420). (**E**) Normalized B-factor of Gc glycoprotein determined using I-TASSER. (**F**) The predicted solvent accessibility of the Gc glycoprotein determined using NetSurfP-3.0.

Gc glycoprotein is the largest of the membrane glycoproteins and along with Gn, forms the envelope of the virus and is crucial for its structural integrity and interaction with host cells. Gc glycoprotein facilitates viral entry into host cells as well as release from host cells. Bioinformatic analysis using Protter demonstrated that the Gc glycoprotein has one transmembrane domain (Figure 2A). The structure was confirmed using Phobius (Figure 2B). AlphaFold 2 predicted the 3D structure of the Gc glycoprotein (Figure 2C). The B-cell and T-cell epitopes are depicted in Figure 2D and Figure S2. Some of the B-cell and T-cell epitopes overlap and may be considered vaccine candidates. The normalized B-factor of the Gc glycoprotein is shown in Figure 2E. The predicted solvent accessibility of the Gc glycoprotein is plotted in Figure 2F. The C-score, TM-score, and RMSD of the Gc glycoprotein are shown in Table 2. The PTM of the Gc glycoprotein is shown in Figure S7.

### 3.4. NSm Protein

**Figure 3:**
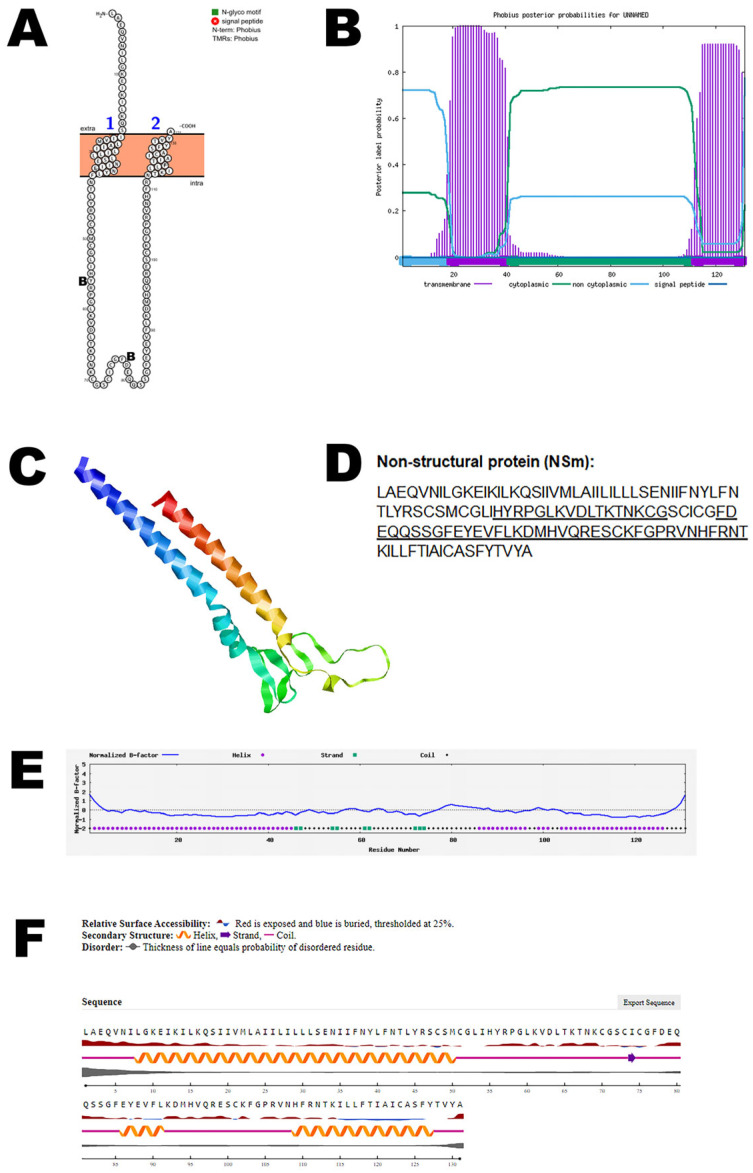
**Predicted structural model of the OROV NSm non-structural protein.** (**A**) The topology of the OROV NSm non-structural protein as determined by Protter. The T-cell (T) and B-cell (B) epitope positions are marked in the figure. (**B**) The topology of the OROV NSm non-structural protein as determined by Phobius. (**C**) The predicted protein structure of the NSm non-structural protein (ribbon diagram) determined using the software AlfaFold 2. (**D**) The B-cell (underlined) of the OROV NSm non-structural protein (Accession UYI36405; Amino acid: 351–481). Score for the T-cell epitope below 0.3, hence not considered. (**E**) Normalized B-factor of NSm protein determined using I-TASSER. (**F**) The predicted solvent accessibility of the NSm protein determined using NetSurfP-3.0.

NSm is a non-structural protein and is encoded in the medium (M) segment of the RNA of OROV. The NSm protein is not essential for virus replication in the mammalian and mosquito cell lines that were evaluated. NSm is also less prone to mutation [12]. NSm protein is thought to play a role in the virus assembly and budding process [44]. Bioinformatic analysis using Protter demonstrated that the NSm protein has two transmembrane domains (Figure 3A). The structure was confirmed using Phobius (Figure 3B). AlphaFold 2 predicted the 3D structure of the NSm protein (Figure 3C). The T-cell epitopes for the NSm protein and B-cell epitopes are depicted in Figure 3D and Figure S3. The normalized B-factor of NSm protein is shown in Figure 3E. The predicted solvent accessibility of the NSm protein is plotted in Figure 3F. The C-score, TM-score, and RMSD of NSm protein are shown in Table 2. The PTM of the protein is shown in Figure S7.

### 3.5. NSs Protein

**Figure 4:**
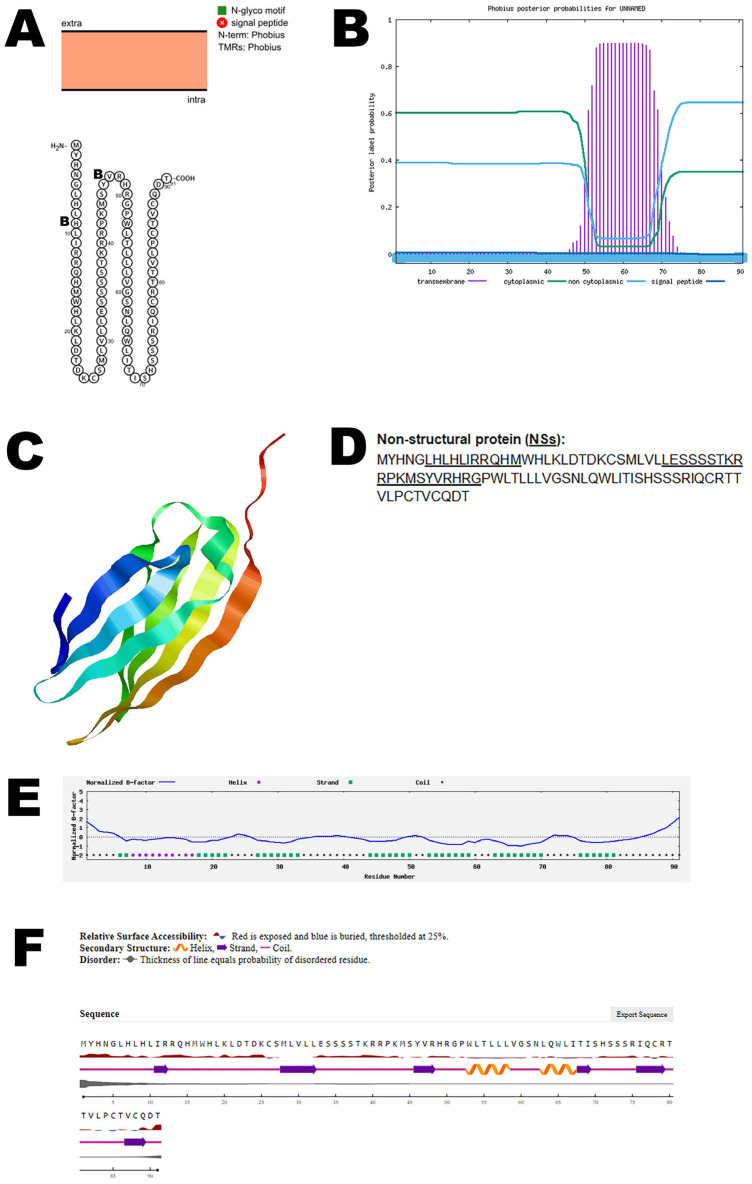
**Predicted structure of the OROV NSs non-structural protein.** (**A**) The topology of the OROV NSs non-structural protein as determined by Protter. The T-cell (T) and B-cell (B) epitope positions are marked in the figure. (**B**) The topology of the OROV NSs non-structural protein as determined by Phobius. (**C**) The predicted protein structure of the NSs non-structural protein (ribbon diagram) determined using the software AlfaFold 2. (**D**) The B-cell (underlined) epitope of OROV NSs non-structural protein (Accession: AEH03002). Score for T-cell epitope below 0.3, hence not considered. (**E**) Normalized B-factor of the NSs protein determined using I-TASSER. (**F**) The predicted solvent accessibility of the NSs protein determined using NetSurfP-3.0.

The small (S) segment of the virus encodes two overlapping open reading frames (ORFs), which are responsible for producing the nucleocapsid protein (N) and the nonstructural protein NSs. The nucleocapsid protein (N) plays a key role in forming the viral core, while the NSs protein has a crucial function in the virus’s ability to evade the host’s immune response. Specifically, NSs acts as a type I interferon inhibitor, effectively suppressing the host’s antiviral defenses. This inhibition of interferon response is vital for the virus, as it enhances viral replication and contributes significantly to the virus’s pathogenicity. Thus, the NSs protein is essential not only for evading immune detection, but also for the overall replication and disease process of the virus [12]. Bioinformatic analysis using Protter demonstrated that the NSs protein has no transmembrane domains (Figure 4A). The structure was confirmed using Phobius (Figure 4B). AlphaFold 2 predicted the 3D structure of the NSs protein (Figure 4C). The NSs T-cell epitopes and B-cell epitopes are depicted in Figure 4D and Figure S4. The normalized B-factor of the NSs protein is shown in Figure 4E. The predicted solvent accessibility of the NSs protein is plotted in Figure 4F. The C-score, TM-score, and RMSD of the NSs protein are shown in Table 2. The PTM of the NSs protein is shown in Figure S7.

### 3.6. Nucleocapsid Protein

**Figure 5:**
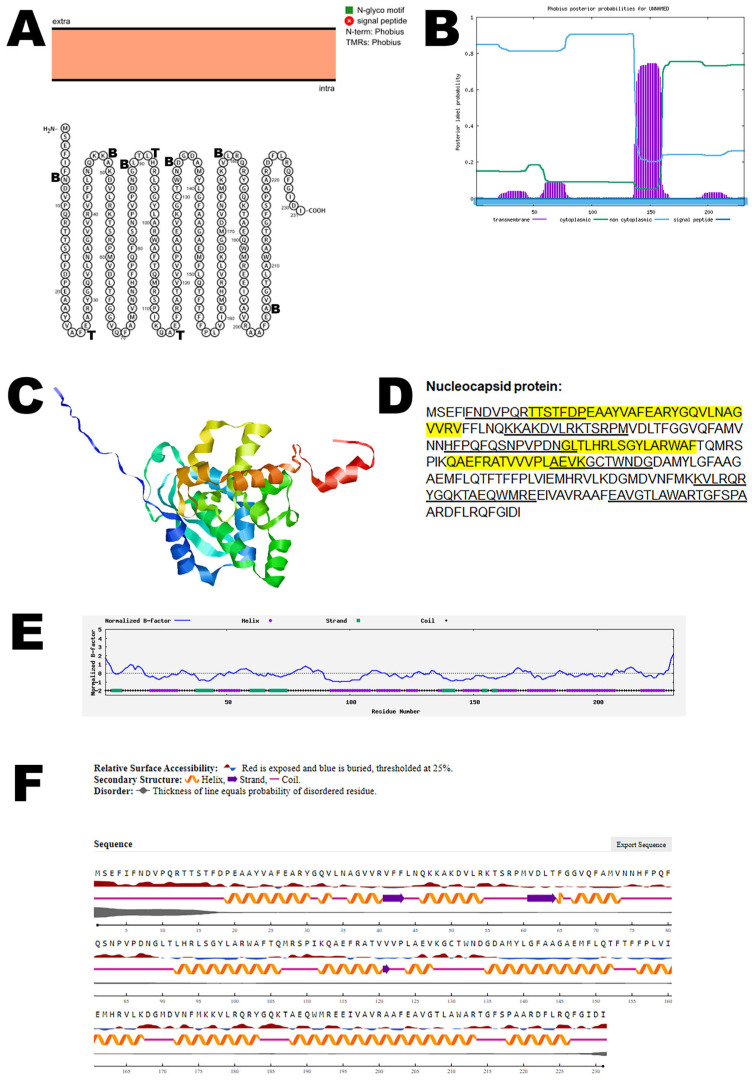
**The predicted structure of the nucleocapsid protein of OROV.** (**A**) The topology of the OROV nucleocapsid protein as determined by Protter. The T-cell (T) and B-cell (B) epitope positions are marked in the figure. (**B**) The topology of the OROV nucleocapsid protein as determined by Phobius. (**C**) The predicted protein structure of the nucleocapsid protein (ribbon diagram) determined using the software AlfaFold 2. (**D**) The B-cell (underlined) and T-cell epitope (yellow shaded) (Score > 0.5) of the OROV nucleocapsid protein (Accession: AJE24680). (**E**) Normalized B-factor of the nucleocapsid protein determined using I-TASSER. (**F**) The predicted solvent accessibility of the nucleocapsid protein determined using NetSurfP-3.0.

This protein encapsulates the viral RNA, forming the nucleocapsid that protects the genetic material and aids in the viral replication process. Protter demonstrated that the nucleocapsid protein has no transmembrane domains (Figure 5A). The structure was confirmed using Phobius (Figure 5B). The predicted 3D structure of the nucleocapsid protein as determined by AlphaFold 2 is shown in Figure 5C. The nucleocapsid protein has both T-cell epitopes and B-cell epitopes that partially overlap and are depicted in Figure 5D and Figure S5. The normalized B-factor of nucleocapsid protein is shown in Figure 5E. The predicted solvent accessibility of nucleocapsid protein is plotted in Figure 5F. The C-score, TM-score, and RMSD of nucleocapsid protein are shown in Table 2. The PTM of the nucleocapsid protein is shown in Figure S7.

### 3.7. RNA Polymerase

**Figure 6:**
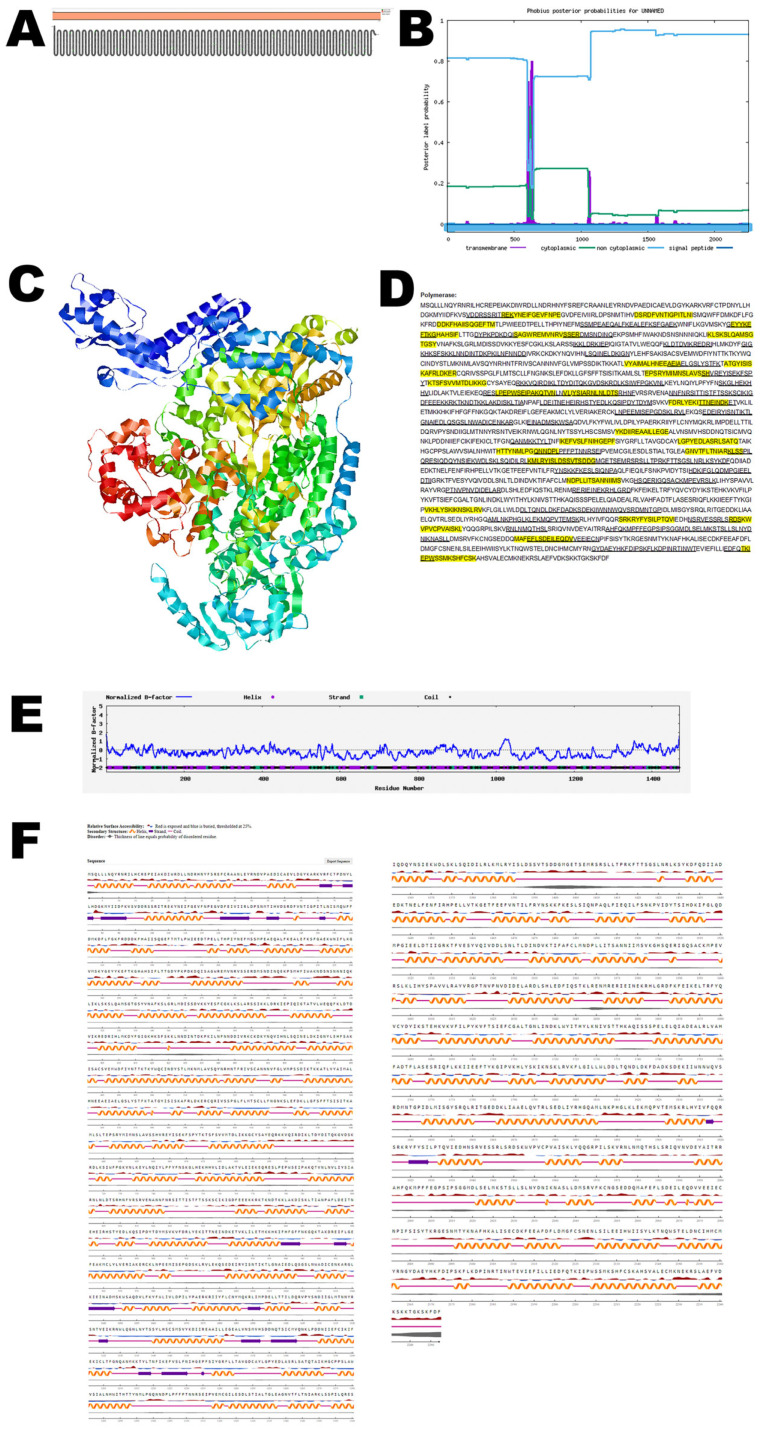
**Predicted structure of the OROV RNA polymerase.** (**A**) The topology of the OROV RNA polymerase as determined by Protter. (**B**) The topology of OROV RNA polymerase as determined by Phobius. (**C**) The predicted protein structure of RNA polymerase (ribbon diagram) determined using the software Phyre 2 (http://www.sbg.bio.ic.ac.uk/phyre2, accessed on 15 August 2024). (**D**) The B-cell (underlined) and T-cell epitope (yellow shaded) (Score > 0.5) of the OROV RNA polymerase (Accession: AJE24678). (**E**) Normalized B-factor of RNA polymerase determined using I-TASSER. (**F**) The predicted solvent accessibility of RNA polymerase determined using NetSurfP-3.0.

This RNA polymerase enzyme is essential for transcribing the viral RNA and is involved in the replication of the virus’s genetic material. The RNA polymerase is the largest protein of OROV. Protter demonstrated that the nucleocapsid protein has no transmembrane domains (Figure 6A). The structure was confirmed using Phobius (Figure 6B). The predicted 3D structure of the nucleocapsid protein as determined by Phyre 2 is shown in Figure 6C. The nucleocapsid protein has both T-cell epitopes and B-cell epitopes that overlap and are depicted in Figure 6D and Figure S6. The normalized B-factor of RNA polymerase is shown in Figure 6E. The predicted solvent accessibility of RNA polymerase is shown in Figure 6F. The C-score, TM-score, and RMSD of RNA polymerase are shown in Table 2. The PTM of RNA polymerase is shown in Figure S7.

### 3.8. Comparative Analysis

To assess whether the protein sequences of OROV possess unique characteristics distinct from other members of the Orthobunyavirus family, we conducted a comparative sequence analysis. The protein sequences of OROV were aligned and evaluated against those of two representative Orthobunyavirus species—Schmallenberg virus (SBV) and Madre de Dios virus (MDDV). This comparison aimed to identify conserved and variable regions, highlighting sequence similarities, potential evolutionary divergence, and unique motifs specific to OROV that may contribute to its distinct biological or pathogenic properties.

Schmallenberg virus (SBV) is an emerging RNA virus that was first identified in cattle in Germany in 2011, belonging to the genus Orthobunyavirus. This virus is closely related to the OROV, another member of the same genus [45]. To better understand their relationship, we conducted a detailed comparison of the protein sequences between SBV and OROV. Our analysis revealed varying degrees of similarity across key viral proteins: the Gn glycoprotein shared a 47.41% sequence identity (Figure S8), while the Gc glycoprotein showed a lower identity of 34.59% (Figure S9). The non-structural proteins NSm and NSs exhibited 28.95% (Figure S10) and 57.14% identity (Figure S11), respectively. Additionally, the nucleocapsid protein displayed the highest similarity at 69.70% (Figure S12), and the RNA polymerase protein showed 58.11% identity between the two viruses (Figure S13). These findings highlight both the genetic divergence and conserved elements within their protein structures, providing insights into their evolutionary relationship and potential functional similarities.

A comparative analysis of the amino acid sequences between the Gn glycoprotein of OROV and MDDV showed a sequence identity of 68.86% (Figure S14) whereas the Gc glycoprotein of OROV showed a sequence identity of 59.47% with the MDDV counterpart (Figure S15). The non-structural protein NSm of OROV had an identity of 54.20% with the MDDV NSm protein (Figure S16), whereas the NSs of OROV and MDDV showed a sequence identity of 91.21% (Figure S17). Interestingly the nucleocapsid protein of both OROV and MDDV was identical (100%), demonstrating that the sequence is highly conserved (Figure S18). The RNA polymerase of OROV and MDDV showed a sequence identity of 94.89% (Figure S19). The findings enhance our understanding of the genetic diversity and evolutionary dynamics within the Orthobunyavirus genus and highlight the important role of genetic reassortment in the emergence of novel viruses.

## 4. Discussion

OROV, or the Oropouche virus, was first detected in 1955 in a febrile forest worker in a village in Trinidad and Tobago called Vega de Oropouche, near the Oropouche River [1]. The virus later gained attention in 1960 when it was detected in a sloth in Brazil, which led to its colloquial name, “sloth fever”. A significant outbreak occurred in Brazil in 1961, resulting in around 11,000 reported cases [46]. Since then, more than 500,000 cases of OROV have been documented across the Americas. However, this number likely underrepresents the true extent of the virus’s impact, as many cases may go unreported or undiagnosed, suggesting that the actual prevalence and transmission of OROV could be considerably higher [47,48].

In June 2024, the first cases of the Oropouche virus disease were reported in Cuba [49]. As of 16 August 2024, the Centers for Disease Control and Prevention (CDC) have reported 21 cases of Oropouche virus disease among U.S. travelers returning from Cuba [6]. In June and July 2024, the EU reported 19 imported cases of Oropouche virus disease for the first time. The Oropouche virus disease involved travel to Cuba and Brazil [50]. In 2024, two deaths were reported due to OROV [51]. Currently, there are no vaccines to protect against OROV. Due to the potential for disease spread driven by climate change and increased international travel for business and tourism, there is a growing need for an effective vaccine to protect against OROV.

Elucidating the protein structure of OROV offers critical insights into the mechanisms of viral entry into human cells and the strategies the virus employs to evade the host immune response. Structural analysis, particularly of the surface glycoproteins involved in host cell entry, help us identify specific sites that are essential for the virus’s function. These sites can become prime targets for vaccine development. By understanding the 3D arrangement of these proteins, we can predict how antibodies might bind to and neutralize the virus, guiding the selection of the most promising vaccine targets.

In this study, we utilized bioinformatic tools to characterize the structural features and predict the antigenic epitopes of OROV proteins. We identified both B-cell and T-cell epitopes of the proteins of OROV. The two known structural proteins of OROV are the membrane glycoproteins Gn and Gc. The Gn and Gc glycoproteins play a crucial role in both viral entry into host cells and the release of newly formed viral particles. Additionally, they are essential for maintaining the structural integrity of the virus. Our bioinformatics analysis revealed that these glycoproteins contain transmembrane domains, which are critical for their function. Furthermore, we identified overlapping B-cell and T-cell epitopes within these glycoproteins, suggesting that they could serve as promising candidates for vaccine development. These epitopes have the potential to stimulate both humoral and cellular immune responses, making Gn and Gc glycoproteins highly suitable targets for protective immunity against the virus.

Both the structural proteins Gn and Gc, along with non-structural proteins, hold significant potential for use in diagnostic applications. The structural proteins Gn and Gc, which are vital for viral entry and assembly, offer specific antigenic sites that can be targeted for accurate detection of the virus in infected individuals. These proteins can serve as biomarkers in serological assays, aiding in the identification of immune responses and the detection of active infections.

In addition to the structural proteins, non-structural proteins, which are involved in the viral replication and modulation of host–cell processes, also present valuable diagnostic targets. These proteins can help in distinguishing between acute and past infections, as they are typically expressed at different stages of the viral life cycle. By incorporating both structural and non-structural proteins into diagnostic tools, more comprehensive and sensitive assays can be developed, improving the ability to detect early infections, monitor disease progression, and assess immune responses in vaccinated or previously exposed individuals. Bioinformatic analysis did not identify any potential T-cell epitopes within the OROV proteins NSm and NSs. This suggests that these proteins may lack regions capable of eliciting a T-cell-mediated immune response.

Adhikari et al. [52], through immunoinformatic analysis, identified several putative T- and B-cell epitopes within the OROV M-segment polyprotein. A recent study reported a promising vaccine candidate based on a replication-competent vesicular stomatitis virus (VSV) vector engineered to express OROV glycoproteins, which demonstrated protective efficacy in a mouse model [53]. The chimeric virus (VSV-OROV) was designed to assess its immunogenic properties and was found to induce a strong neutralizing antibody response, with much of the activity directed toward the N-terminal region of the structural Gc protein. Importantly, vaccination with VSV-OROV significantly reduced the viral loads in mice challenged with the wild-type OROV strain. The B-cell epitope identified in this study was found to be identical to the one previously characterized by Adhikari et al. [52] and Stubbs et al. [53]. This consistency across multiple investigations reinforces the reliability of the epitope’s localization and its potential significance in immune recognition. Such concordance suggests that this specific epitope plays a crucial and conserved role in antigen–antibody interactions, making it a promising target for further immunological studies and potential vaccine or diagnostic development.

Fold stability plays a critical role in determining the availability and accessibility of B-cell epitopes. Properly folded protein structures are essential for maintaining the native conformation of epitopes, which in turn influences how effectively they are recognized by B-cell receptors [54]. In our analysis of OROV proteins, we observed that the majority of predicted B-cell and T-cell epitopes were localized on the folded regions of the proteins, as illustrated by the Protter topology maps. These exposed epitopes, prominently displayed on the protein surface, are more likely to be accessible to immune surveillance mechanisms. Their strategic positioning on the protein fold suggests a higher potential for immunogenicity, as surface-exposed regions are more readily available for interaction with B-cell receptors and antigen-presenting cells. This highlights the importance of considering protein structural integrity when identifying candidate epitopes for vaccine or diagnostic development. Ensuring that these epitopes remain structurally stable and exposed in their native context could significantly enhance the immune response against OROV.

Our findings provide a foundational framework for developing more precise and effective diagnostic assays by targeting specific immunogenic regions of the virus. The conserved B-cell epitopes represent a promising direction for rapid diagnostic test development. Because these epitopes are stable and shared across multiple strains or variants, they offer a reliable target for consistent antibody recognition. Identifying such epitopes could help design sensitive and specific assays that minimize cross-reactivity while maintaining broad applicability. Furthermore, diagnostics built on conserved B-cell epitopes may remain effective even as pathogens evolve, thereby reducing the need for frequent redesigns [55]. Moreover, these epitopes offer promising candidates for the development of vaccines that can elicit strong and targeted immune responses, thereby enhancing protection against OROV infection. The study not only advances our understanding of OROV’s molecular biology, but also supports the development of innovative preventive and diagnostic strategies tailored to the virus’s immunological profile.

Post-translational modifications (PTMs) play a critical role in determining the stability, functionality, and therapeutic potential of many proteins and peptides used in medicine [56]. PTMs refer to the covalent addition of small molecules, chemical groups, or macromolecules—such as phosphate groups, carbohydrates, lipids, or even small proteins like ubiquitin—to specific amino acid residues of a protein after its synthesis. These modifications can profoundly influence protein folding, localization, activity, and interactions.

During viral infections, host cells often employ PTMs as part of their antiviral defense mechanisms. These modifications regulate host proteins involved in immune signaling pathways, helping to detect and limit viral replication. For example, PTMs can activate key immune sensors, inhibit the synthesis of viral proteins, and promote the degradation of viral components, all contributing to the eventual clearance of the virus from the host.

Conversely, viruses can exploit PTMs to their advantage. Modifications to viral proteins can enhance their solubility, stability, and ability to evade host immune responses. PTMs also contribute to increased virulence and immunogenicity, making them important targets for therapeutic intervention [57].

In the case of OROV, the present study shows that the Gc glycoprotein and viral RNA-dependent RNA polymerase undergo extensive post-translational modification compared with other viral proteins (Figure S7). The Gc glycoprotein, a structural component of the viral envelope, is particularly notable for its high level of PTMs. These modifications may contribute to its role in host–cell attachment and membrane fusion, making it a promising candidate for vaccine development due to its immunogenic properties and potential to elicit a strong protective immune response.

In a mouse model of ehrlichiosis, we analyzed the post-translational modifications (PTMs) of antigenic proteins from *Ehrlichia muris* and Ixodes ovatus Ehrlichia (IOE). The antigenic proteins of *E. muris* exhibited a greater number of PTMs compared with those of IOE. Mice infected with *E. muris* survived, while infection with IOE was fatal. Notably, the post-translationally modified proteins Hsp60 and P28-19 also demonstrated potential as vaccine candidates [58,59].

Fatalities caused by OROV infection are relatively rare compared with those associated with many other viral pathogens. Despite its typically non-lethal nature, OROV is capable of efficient replication in non-human primates and has been shown to elicit strong immune responses, indicating its immunostimulatory properties [60]. These findings suggest that host immune recognition plays a critical role in controlling OROV infection. Based on this, we hypothesize that PTMs of OROV proteins may contribute to the activation of protective immune mechanisms. Such PTMs could enhance antigen presentation or modulate protein structure in a way that facilitates viral clearance, offering potential targets for vaccine or therapeutic development.

To investigate whether OROV possesses unique protein characteristics compared with other Orthobunyaviruses, a comparative sequence analysis was performed. Protein sequences from OROV were aligned with those of two representative species within the genus: Schmallenberg virus (SBV) and Madre de Dios virus (MDDV). The analysis revealed that while the structural proteins of OROV and SBV exhibited low sequence identity, the non-structural proteins showed relatively higher similarity. These differences may suggest functional divergence in viral components related to structure, while conservation in non-structural proteins could reflect shared mechanisms in replication or host interaction.

Genetic reassortment is a well-established mechanism of evolution among orthobunyaviruses, particularly those within the same serogroup, occurring when a host is simultaneously infected by multiple virus strains. This exchange of genome segments can give rise to novel genotypes with altered phenotypic traits such as increased virulence or expanded host range. OROV has been implicated as a parental virus in several natural reassortment events, with MDDV being a notable example—harboring genomic segments derived from both OROV and other orthobunyaviruses. These findings illustrate the dynamic nature of orthobunyavirus evolution and emphasize the importance of reassortment in the emergence of potentially pathogenic viral variants [61]. The analysis revealed that while the structural proteins of OROV and MDDV exhibited low sequence identity, the non-structural proteins showed relatively higher similarity.

Contemporary bioinformatics tools such as AlphaFold and I-TASSER have revolutionized structural biology by enabling the rapid prediction of protein structures from amino acid sequences. However, despite their capabilities, these predictive models come with inherent limitations that must be carefully considered. Structural predictions are based on computational algorithms that may not fully capture the dynamic and complex nature of protein folding, particularly in regions of intrinsic disorder, flexible loops, or multi-domain interactions. This uncertainty becomes especially critical when predicted structures are used to infer functional or immunological properties—for instance, identifying active sites, binding interfaces, or antigenic epitopes. Even minor inaccuracies in structural predictions can lead to incorrect assumptions about a protein’s mechanism of action, interaction partners, or immunogenic potential. As a result, while AlphaFold and I-TASSER serve as powerful tools for hypothesis generation and provide valuable preliminary insights, their outputs should not be interpreted as definitive. Experimental validation—through methods such as X-ray crystallography, cryo-electron microscopy, NMR spectroscopy, or functional assays—remains essential to verify the accuracy and biological relevance of the predicted structures. Only through such validation can researchers ensure that downstream analyses are built on reliable foundations, thereby avoiding potentially misleading conclusions.

In our analysis of OROV proteins, the predicted structural models consistently showed lower C-scores and TM-scores, along with higher RMSD values, when compared with typical high-confidence protein structures. These metrics collectively indicate reduced confidence in the accuracy and reliability of the predicted models. Such outcomes reflect the inherent challenges of modeling viral proteins, especially in the absence of close structural homologs.

One major limitation stems from the lack of homologous templates in current structural databases. Many OROV proteins do not have well-characterized counterparts with known crystal or NMR structures, which weakens the performance of template-based prediction methods. Since these approaches rely heavily on structural similarity to known proteins, the absence of suitable templates reduces the predictive accuracy, resulting in lower C-scores and unreliable folds.

In addition, many viral proteins exhibit high structural variability. Proteins involved in host interaction or immune evasion often contain intrinsically disordered regions or flexible domains, which do not adopt stable conformations under physiological conditions. These features are notoriously difficult to model using current computational methods, further lowering the confidence in predicted structures.

Another factor affecting prediction quality is the small size or atypical folding patterns of several OROV proteins. For instance, viral glycoproteins often possess extensive post-translational modifications, such as glycosylation, which are not typically accounted for in structural modeling. Such modifications can significantly alter folding and surface properties, leading to inaccuracies in both global and local structure predictions.

Despite these limitations, the structural models—even with lower confidence scores—remain valuable tools for hypothesis generation and experimental planning. They can help identify functional regions such as surface-exposed loops, conserved domains, or potential epitopes relevant for antiviral drug development or vaccine design. Ultimately, integrating computational predictions with experimental techniques like cryo-EM, X-ray crystallography, or NMR spectroscopy will be essential to produce high-resolution, functionally relevant structures of OROV proteins.

## Figures and Tables

**Table 1:** The Oropouche virus (OROV) major proteins, accession numbers, and its function.

OROV Protein	Accession Number (Amino Acid)	Function
Gn glycoprotein	UYI36405 (1–350)	Viral entry and exit
Gc glycoprotein	UYI36405 (482–1420)	Interaction with host cells
NSm non-structural protein	UYI36405 (351–481)	Virus assembly and budding
NSs non-structural protein	AEH03002	Evade host immunity
Nucleocapsid protein	AJE24680	Encapsulates viral RNA
RNA polymerase	AJE24678	Replication

**Table 2:** The C-score, TM-score, and RMSD of OROV virus proteins.

Protein	C-Score	TM-Score	RMSD
Gn glycoprotein	−2.21	0.45 ± 0.15	11.7 ± 4.5 Å
Gc glycoprotein	−2.85	0.39 ± 0.13	16.1 ± 3.1 Å
NSm non-structural protein	−4.30	0.26 ± 0.08	14.7 ± 3.6 Å
NSs non-structural protein	−3.25	0.35 ± 0.12	11.0 ± 4.6 Å
Nucleocapsid protein	1.57	0.93 ± 0.06	2.6 ± 1.9 Å
RNA polymerase	0.52	0.78 ± 0.09	8.6 ± 4.5 Å

## Data Availability

The programs used to generate data are stated in the Section 2: Materials and Methods. The complete data generated in the study is shown in the Section 3: Results.

## Supplementary Materials

The following supporting information can be downloaded at: https://www.mdpi.com/article/10.3390/v17111434/s1. Figure S1: The T-cell epitopes (MHC-I: HLA B35:01)) (Score > 0.3) (underlined), (MHC-II: HLA-DQA1*01:01/DQB1*02:01) (Score > 0.02) (yellow shading) of OROV Gn glycoprotein (Accession: UYI36405; Amino acid: 1–350); Figure S2: The T-cell epitopes (MHC-I: HLA B35:01)) (Score > 0.5) (underlined), (MHC-II: HLA-DQA1*01:01/DQB1*02:01) (Score > 0.04) (yellow shading) of OROV Gc glycoprotein (Accession: UYI36405; Amino acid: 482–1420); Figure S3: The T-cell epitopes (MHC-I: HLA B35:01) (Score > 0.11) (underlined), (MHC-II: HLA-DQA1*01:01/DQB1*02:01) (Score > 0.01) (yellow shading) of OROV NSm protein (Accession: UYI36405; Amino acid: 351–481); Figure S4: The T-cell epitopes (MHC-I: HLA B35:01) (Score > 0.11) (underlined), (MHC-II: HLA-DQA1*01:01/DQB1*02:01) (Score > 0.005) (yellow shading) of OROV NSs protein (Accession: AEH03002); Figure S5: The T-cell epitopes (MHC-I: HLA B35:01) (Score > 0.5) (underlined), (MHC-II: HLA-DQA1*01:01/DQB1*02:01) (Score > 0.03) (yellow shading) of OROV nucleocapsid protein (Accession: AJE24680); Figure S6: The T-cell epitopes (MHC-I: HLA B35:01) (Score > 0.43) (underlined), (MHC-II: HLA-DQA1*01:01/DQB1*02:01) (Score > 0.01) (yellow shading) of OROV RNA polymerase (Accession: AJE24678); Figure S7: Post-translational modifications (PTMs) of OROV proteins are color-coded by amino acid; Figure S8: Alignment of Gn glycoprotein of OROV (Accession UYI36405) and Schmallenberg virus (SBV) (Accession: YP_009666912.1) shows a percentage identity of 47.41%; Figure S9: Alignment of Gc glycoprotein of OROV (Accession: UYI36405) and SBV (Accession: YP_009666912.1) shows a percentage identity of 34.59%; Figure S10: Alignment of NSm of OROV (Accession: UYI36405) and SBV (Accession: YP_009666912.1) shows a percentage identity of 28.95%; Figure S11: Alignment of NSs of OROV (Accession: AEH03002.1) and SBV (Accession: XCH39114.1) shows a percentage identity of 57.14%; Figure S12: Alignment of nucleocapsid protein of OROV (Accession: AJE24680) and SBV (Accession: XCH39113) shows a percentage identity of 69.70%; Figure S13: Alignment of RNA polymerase of OROV (Accession: AJE24678) and SBV (Accession: YP_009666911.1) shows a percentage identity of 58.11%; Figure S14: Alignment of Gn glycoprotein of OROV (Accession: UYI36405) and Madre de Dios virus (MDDV) (Accession: AIK67310) shows a percentage identity of 68.86%; Figure S15: Alignment of Gc glycoprotein of OROV (Accession: UYI36405) and MDDV (Accession: AIK67310) shows a percentage identity of 59.47%; Figure S16: Alignment of non-structural protein NSm of OROV (Accession: UYI36405) and MDDV (Accession: AIK67310) shows a percentage identity of 54.20%; Figure S17: Alignment of non-structural protein NSs of OROV (Accession: AEH03002) and MDDV (Accession: AIK67309) shows a percentage identity of 91.21%; Figure S18: Alignment of nucleocapsid protein of OROV (Accession AJE24680) and MDDV (Accession AHY22351) shows a percentage identity of 100%; Figure S19: Alignment of RNA polymerase of OROV (Accession AJE24678) and MDDV (Accession AIK67311) shows a percentage identity of 94.89%.
