# Supplementary material for: Charting the Proteins of Oropouche Virus
Source: Viruses. 2025 Oct 28;17(11):1434. doi: 10.3390/v17111434 (PMC12656799; doi:10.3390/v17111434)

SUPPLEMENTARY FIGURES

Figure S1. The T-cell epitopes (MHC-I: HLA B35:01)) (Score>0.3) (underlined), (MHC-II: HLA-DQA1*01:01/DQB1*02:01) (Score>0.02) (yellow shading) of OROV Gn glycoprotein (Accession: UYI36405; Amino acid:1-350).

MANLIIISMILGIAYGHPLSTSQIGDRCFAGGSLFKEMNLSVGLGEICVKDDISIVKSTTAFSKNALA
LEATTKFYRSFIVKDWSECNPVLDKFGNFMVLNVDDNGHLVPKMYTCRAACDIRLNKDNAEIIL
SSTKLNHFEIVGTTSTSGWFKNTITNNLEHTCEHVTVNCGQKSVKFHACFRQHRSCIRFFKGTYM
PYSMIEAMCVNIELIILTLYIFAAIIFALIITKSYIAYLLLPLFYPVTWLYGKVYKRINSCPNCLLASHP
FTSCPKICICGSRFSCTEALKVHRMGKDCLGYKSLSKARQMCKSKSWSFTAAILTGLILMEFVSPIA
GERMYKLEELADDYIE

Figure S2. The T-cell epitopes (MHC-I: HLA B35:01)) (Score>0.5) (underlined), (MHC-II: HLA-DQA1*01:01/DQB1*02:01) (Score>0.04) (yellow shading) of OROV Gc glycoprotein (Accession: UYI36405; Amino acid: 482-1420).

DEDCLSKNIRITYQELHSCIGPHIMGDTCMSKSELYSDLLSKNLITEYDKKYFEPDTVNDQFNKIEF
AQDAHRMILLERILYKTECEMLSLKKNSGPYNVAWRTYLKNHNIDLCSRHNYKMICQCINTHSM
CKNTDIDFNKEIETYYKANAAAYRSDFNTIIDTLKTAFRGLTKVLIENYIEKDDSDALKALFSNISD
SVQNNYQMVGVLKFASKLLNISLGRTTRSAQHSIMTNEIPKSNPFTDYSYSSVNIKECMSPESLKCF
KKRDSAPHTNHLLCKIDNKYKAFDWPEIETVQKGQKLCLGDSHCNLEFTAITADKIMSLTNCYK
ESFTAQPADMQTGIKKCSADEIGECMTLEDKSWPIVFCGDKYYYSEGKEHAKDGSINNYCLANK
CSEQRFPIHKNWFKKCNWDKTHKEFTTMRQINYNDITSYRKAIESEIGTDLMTHHYKPTKNLPH
VVPRYHSIDVQGTESTEGIINGFIQNTIPAISGLGVGYHLGFKGNQLFDIVIFVKKAVYKAQYQKVY
TTGPSISINIEHNEKCTGHCPEKIPAKEGWLTFSKEHTSSWGCEEYGCLAIDTGCLYGSCQDVIRPE
LDIYKKIGSEASLIEICITLPHETYCNDMDILEPIIGDKLSASFQNTQTNQLPNLMAYKKGKVYTGQI
NDVGNTALQCGSIQVVNGSTIGSGNPKFDYICHAMRRKDVIVRKCFNDNYQSCTRLNPRNDLIP
YRKGDIIEISKTGSNMGQMTFKIELGDINYKIFTKSVDLQMSGVCAGCIDCAEGISCSINADVSAET
VCHCKTNCEDFISNIVISPQIKTYNIKVHCKSKVEKITANICGRNIDLQLTVKPYNQKIDLSQLDES
NYIKEEDLQCGTWLCKVQKEGIDIVFKGLFSGLGKYWAILIYSIIGVIVVILLIYVLLPIGRLLKAFLIK
NEIEYTMEQKIK

Figure S3. The T-cell epitopes (MHC-I: HLA B35:01) (Score>0.11) (underlined), (MHC-II: HLA-DQA1*01:01/DQB1*02:01) (Score>0.01) (yellow shading) of OROV NSm protein (Accession: UYI36405; Amino acid: 351-481).

LAEQVNILGKEIKILKQSIIVMLAIILILLLSENIIFNYLFNTLYRSCSMCGLIHYRPGLKVDLTKTNKC
GSCICGFDEQQSSGFEYEVFLKDMHVQRESCKFGPRVNHFRNTKILLFTIAICASFYTVYA

Figure S4. The T-cell epitopes (MHC-I: HLA B35:01) (Score>0.11) (underlined), (MHC-II: HLA-DQA1*01:01/DQB1*02:01) (Score>0.005) (yellow shading) of OROV NSs protein (Accession: AEH03002).

MYHNGLHLHLIRRQHMWHLKLDTDKCSMLVLLESSSSTKRRPKMSYVRHRGPWLTLLLVGSNL
QWLITISHSSSRIQCRTTVLPCTVCRDT

Figure S5. The T-cell epitopes (MHC-I: HLA B35:01) (Score>0.5) (underlined), (MHC-II: HLA-DQA1*01:01/DQB1*02:01) (Score>0.03) (yellow shading) of OROV nucleocapsid protein (Accession: AJE24680).

MSEFIFNDVPQRTTSTFDPEAAYVAFEARYGQVLNAGVVRVFFLNQKKAKDVLRKTSRPMVDLT
FGGVQFAMVNNHFPQFQSNPVPDNGLTLHRLSGYLARWAFTQMRSPIKQAEFRATVVVPLAEV
KGCTWNDGDAMYLGFAAGAEMFLQTFTFFPLVIEMHRVLKDGMDVNFMKKVLRQRYGQKTA
EQWMREEIVAVRAAFEAVGTLAWARTGFSPAARDFLRQFGIDI

Figure S6. The T-cell epitopes (MHC-I: HLA B35:01) (Score>0.43) (underlined), (MHC-II: HLA-DQA1*01:01/DQB1*02:01) (Score>0.01) (yellow shading) of OROV RNA polymerase (Accession: AJE24678).

MSQLLLNQYRNRILHCREPEIAKDIWRDLLNDRHNYFSREFCRAANLEYRNDVPAEDICAEVLD
GYKARKVRFCTPDNYLLHDGKMYIIDFKVSVDDRSSRITREKYNEIFGEVFNPEGVDFEIVIIRLDPS
NMTIHVDSRDFVNTIGPITLNISMQWFFDMKDFLFGKFRDDDKFHAIISQGEFTMTLPWIEEDTPE
LLTHPIYNEFMSSMPEAEQALFKEALEFKSFGAEKWNIFLKGVMSKYGEYYKEFTKGHAHSIFLTT
GDYPKPDKDQISAGWREMVNRVSSERDMSNDINQEKPSMHFIWAKNDSNSNNNIQKLIKLSKSL
QAMSGTGSYVNAFKSLGRLMDISSDVKKYESFCGKLKSLARSSIKKLDRKIEPIQIGTATVLWEQQF
KLDTDVIKREDRIHLMKDYFGIGKHKSFSKKLNNDINTDKPKILNFNNDDIVRKCKDKYNQVIH
NLSQINELDKIGNYLEHFSAKISACSVEMWDFIYNTTKTKYWQCINDYSTLMKNMLAVSQYNRH
NTFRIVSCANNNVFGLVMPSSDIKTKKATLVYAIMALHNEEAEIAELGSLYSTFKTATGYISISKAF
RLDKERCQRIVSSPGLFLMTSCLLFNGNKSLEFDKLLGFSFFTSISITKAMLSLTEPSRYMIMNSLAV
SSHVREYISEKFSPYTKTSFSVVMTDLIKKGCYSAYEQRKKVQIRDIKLTDYDITQKGVDSKRDLKSI
WFPGKVNLKEYLNQIYLPFYFNSKGLHEKHHVLIDLAKTVLEIEKEQRESLPEPWSEIPAKQTVNL
NVLIYSIARNLNLDTSRHNFVRSRVENANNFNRSITTISTFTSSKSCIKIGDFEEEKKRKTKNDTKKL
AKDISKLTIANPAFLDEITNEHEIRHSTYEDLKQSIPDYTDYMSVKVFDRLYEKITTNEINDKETVKL
ILETMKKHKIFHFGFFNKGQKTAKDREIFLGEFEAKMCLYLVERIAKERCKLNPEEMISEPGDSKL
RVLEKQSEDEIRYISNTIKTLGNAIEDLQSGSLNWADICENKARGLKIEINADMSKWSAQDVLFKY
FWLIVLDPILYPAERKRIIYFLCNYMQKRLIMPDELLTTILDQRVPYSNDIIGLMTNNYRSNTVEIKR
NWLQGNLNYTSSYLHSCSMSVYKDIIREAAILLEGEALVNSMVHSDDNQTSICMVQNKLPDDNII
EFCIKIFEKICLTFGNQANMKKTYLTNFIKEFVSLFNIHGEPFSIYGRFLLTAVGDCAYLGPYEDLAS
RLSATQTAIKHGCPPSLAWVSIALNHWITHTTYNMLPGQNNDPLPFFPTNNRSEIPVEMCGILES
DLSTIALTGLEAGNVTFLTNIARKLSSPILQRESIQDQYNSIEKWDLSKLSQIDILRLKMLRYISLDSS
VTSDDGMGETSEMRSRSLLTPRKFTTSGSLNRLKSYKDFQDIIADEDKTNELFENFIRHPELLVTKG
ETFEEFVNTILFRYNSKKFKESLSIQNPAQLFIEQILFSNKPVIDYTSIHDKIFGLQDMPGIEELDTIIG
RKTFVESYVQIVDDLSNLTLDINDVKTIFAFCLMNDPLLITSANNIIMSVKGHSQERIGQSACKMPE
VRSLKLIHYSPAVVLRAYVRGPTNVPNVDIDELARDLSHLEDFIQSTKLRENMRERIEINEKRHLG
RDFKFEIKELTRFYQVCYDYIKSTEHKVKVFILPYKVFTSIEFCGALTGNLINDKLWYITHYLKNIVS
TTHKAQISSSPELELQIADEALRLVAHFADTFLASESRIQFLKKIIEEFTYKGIPVKHLYSKIKNSKLR
VKFLGILLWLDDLTQNDLDKFDADKSDEKIIWNNWQVSRDMNTGPIDLMISGYSRQLRITGEDD
KLIAAELQVTRLSEDLIYRHGQAMLNKPHGLKLEKMQPVTEMSKRLHYIVFQQRSRKRYFYSILPT
QVIEDHNSRVESSRLSRDSKWVPVCPVAISKLYQQGRPILSKVRNLNMQTHSLSRIQVNVDEYAIT
RRAHFQKMPFFEGPSIPSGGMDLSELMKSTSLLSLNYDNIKNASLLDMSRVFKCNGSEDDQMAFE
FLSDEILEQDVVEEIECNPIFSISYTKRGESNMTYKNAFHKALISECDKFEEAFDFLDMGFCSNENLS
ILEEIHWIISYLKTNQWSTELDNCIHMCMYRNGYDAEYHKFDIPSKFLKDPINRTINWTEVIEFILLI
EDFQTKIEPWSSMKSHFCSKAHSVALECMKNEKRSLAEFVDKSKKTGKSKFDF

Figure S7. Post-translational modifications (PTMs) of OROV proteins are color-coded by amino acid. If an amino acid undergoes multiple PTMs, it is underlined in each corresponding color. The PTMs depicted are: K: Methylation; K: Ubiquitination; N: N-linked Glycosylation; S,T: O-linked Glycosylation; C: S-Palmitoylation; K: Formylation; C: S-nitrosylation; S, T, Y: Phosphorylation; K: Sumoylation.

Gn Glycoprotein:

MANLIIISMILGIAYGHPLSTSQIGDRCFAGGSLFKEMNLSVGLGEICVKDDISIVKSTTAFSKNA
LALEATTKFYRSFIVKDWSECNPVLDKFGNFMVLNVDDNGHLVPKMYTCRAACDIRLNKDN
AEIILSSTKLNHFEIVGTTSTSGWFKNTITNNLEHTCEHVTVNCGQKSVKFHACFRQHRSCIRFFK
GTYMPYSMIEAMCVNIELIILTLYIFAAIIFALIITKSYIAYLLLPLFYPVTWLYGKVYKRINSCPNCLL
ASHPFTSCPKICICGSRFSCTEALKVHRMGKDCLGYKSLSKARQMCKSKSWSFTAAILTGLILMEF
VSPIAGERMYKLEELADDYIE

NSm:

LAEQVNILGKEIKILKQSIIVMLAIILILLLSENIIFNYLFNTLYRSCSMCGLIHYRPGLKVDLTKTNK
CGSCICGFDEQQSSGFEYEVFLKDMHVQRESCKFGPRVNHFRNTKILLFTIAICASFYTVYA

Gc Glycoprotein:

DEDCLSKNIRITYQELHSCIGPHIMGDTCMSKSELYSDLLSKNLITEYDKKYFEPDTVNDQFN
KIEFAQDAHRMILLERILYKTECEMLSLKKNSGPYNVAWRTYLKNHNIDLCSRHNYKMICQ
CINTHSMCKNTDIDFNKEIETYYKANAAAYRSDFNTIIDTLKTAFRGLTKVLIENYIEKDDSDALK
ALFSNISDSVQNNYQMVGVLKFASKLLNISLGRTTRSAQHSIMTNEIPKSNPFTDYSYSSV
NIKECMSPESLKCFKKRDSAPHTNHLLCKIDNKYKAFDWPEIETVQKGQKLCLGDSHCNLEFT
AITADKIMSLTNCYKESFTAQPADMQTGIKKCSADEIGECMTLEDKSWPIVFCGDKYYYSEGKEH
AKDGSINNYCLANKCSEQRFPIHKNWFKKCNWDKTHKEFTTMRQINYNDITSYRKAIESEIG
TDLMTHHYKPTKNLPHVVPRYHSIDVQGTESTEGIINGFIQNTIPAISGLGVGYHLGFKGNQLFDI
VIFVKKAVYKAQYQKVYTTGPSISINIEHNEKCTGHCPEKIPAKEGWLTFSKEHTSSWGCEEYG
CLAIDTGCLYGSCQDVIRPELDIYKKIGSEASLIEICITLPHETYCNDMDILEPIIGDKLSASFQNTQT
NQLPNLMAYKKGKVYTGQINDVGNTALQCGSIQVVNGSTIGSGNPKFDYICHAMRRKDVIVR
KCFNDNYQSCTRLNPRNDLIPYRKGDIIEISKTGSNMGQMTFKIELGDINYKIFTKSVDLQMSGVC
AGCIDCAEGISCSINADVSAETVCHCKTNCEDFISNIVISPQIKTYNIKVHCKSKVEKITANICGR
NIDLQLTVKPYNQKIDLSQLDESNYIKEEDLQCGTWLCKVQKEGIDIVFKGLFSGLGKYWAILIY
SIIGVIVVILLIYVLLPIGRLLKAFLIKNEIEYTMEQKIK

NSs:

MYHNGLHLHLIRRQHMWHLKLDTDKCSMLVLLESSSSTKRRPKMSYVRHRGPWLTLLLVGSNL
QWLITISHSSSRIQCRTTVLPCTVCRDT

Nucleocapsid protein:

MSEFIFNDVPQRTTSTFDPEAAYVAFEARYGQVLNAGVVRVFFLNQKKAKDVLRKTSRPMVDL
TFGGVQFAMVNNHFPQFQSNPVPDNGLTLHRLSGYLARWAFTQMRSPIKQAEFRATVVVPLAE
VKGCTWNDGDAMYLGFAAGAEMFLQTFTFFPLVIEMHRVLKDGMDVNFMKKVLRQRYGQKT
AEQWMREEIVAVRAAFEAVGTLAWARTGFSPAARDFLRQFGIDI

RNA polymerase:

MSQLLLNQYRNRILHCREPEIAKDIWRDLLNDRHNYFSREFCRAANLEYRNDVPAEDICAEVLD
GYKARKVRFCTPDNYLLHDGKMYIIDFKVSVDDRSSRITREKYNEIFGEVFNPEGVDFEIVIIRLD
PSNMTIHVDSRDFVNTIGPITLNISMQWFFDMKDFLFGKFRDDDKFHAIISQGEFTMTLPWIEED
TPELLTHPIYNEFMSSMPEAEQALFKEALEFKSFGAEKWNIFLKGVMSKYGEYYKEFTKGHA
HSIFLTTGDYPKPDKDQISAGWREMVNRVSSERDMSNDINQEKPSMHFIWAKNDSNSNNNIQ
KLIKLSKSLQAMSGTGSYVNAFKSLGRLMDISSDVKKYESFCGKLKSLARSSIKKLDRKIEPIQI
GTATVLWEQQFKLDTDVIKREDRIHLMKDYFGIGKHKSFSKKLNNDINTDKPKILNFNNDDIV
RKCKDKYNQVIHNLSQINELDKIGNYLEHFSAKISACSVEMWDFIYNTTKTKYWQCINDYSTL
MKNMLAVSQYNRHNTFRIVSCANNNVFGLVMPSSDIKTKKATLVYAIMALHNEEAEIAELGSL
YSTFKTATGYISISKAFRLDKERCQRIVSSPGLFLMTSCLLFNGNKSLEFDKLLGFSFFTSISITKAM
LSLTEPSRYMIMNSLAVSSHVREYISEKFSPYTKTSFSVVMTDLIKKGCYSAYEQRKKVQIRDIKLT
DYDITQKGVDSKRDLKSIWFPGKVNLKEYLNQIYLPFYFNSKGLHEKHHVLIDLAKTVLEIEKE
QRESLPEPWSEIPAKQTVNLNVLIYSIARNLNLDTSRHNFVRSRVENANNFNRSITTISTFTSSKSCI
KIGDFEEEKKRKTKNDTKKLAKDISKLTIANPAFLDEITNEHEIRHSTYEDLKQSIPDYTDY
MSVKVFDRLYEKITTNEINDKETVKLILETMKKHKIFHFGFFNKGQKTAKDREIFLGEFEAKMCL
YLVERIAKERCKLNPEEMISEPGDSKLRVLEKQSEDEIRYISNTIKTLGNAIEDLQSGSLNWADICE
NKARGLKIEINADMSKWSAQDVLFKYFWLIVLDPILYPAERKRIIYFLCNYMQKRLIMPDELLTTIL
DQRVPYSNDIIGLMTNNYRSNTVEIKRNWLQGNLNYTSSYLHSCSMSVYKDIIREAAILLEGEAL
VNSMVHSDDNQTSICMVQNKLPDDNIIEFCIKIFEKICLTFGNQANMKKTYLTNFIKEFVSLFNIH
GEPFSIYGRFLLTAVGDCAYLGPYEDLASRLSATQTAIKHGCPPSLAWVSIALNHWITHTTYNM
LPGQNNDPLPFFPTNNRSEIPVEMCGILESDLSTIALTGLEAGNVTFLTNIARKLSSPILQRESIQ
DQYNSIEKWDLSKLSQIDILRLKMLRYISLDSSVTSDDGMGETSEMRSRSLLTPRKFTTSGSLN
RLKSYKDFQDIIADEDKTNELFENFIRHPELLVTKGETFEFVNTILFRYNSKKFKESLSIQNPAQLFI
EQILFSNKPVIDYTSIHDKIFGLQDMPGIEELDTIIGRKTFVESYVQIVDDLSNLTLDINDVKTIFAF
CLMNDPLLITSANNIIMSVKGHSQERIGQSACKMPEVRSLKLIHYSPAVVLRAYVRGPTNVPNV
DIDELARDLSHLEDFIQSTKLRENMRERIEINEKRHLGRDFKFEIKELTRFYQVCYDYIKSTEHKV
KVFILPYKVFTSIEFCGALTGNLINDKLWYITHYLKNIVSTTHKAQISSSPELELQIADEALRLVA
HFADTFLASESRIQFLKKIIEEFTYKGIPVKHLYSKIKNSKLRVKFLGILLWLDDLTQNDLDKFDAD
KSDEKIIWNNWQVSRDMNTGPIDLMISGYSRQLRITGEDDKLIAAELQVTRLSEDLIYRHGQAM

LNKPHGLKLEKMQPVTEMSKRLHYIVFQQRSRKRYFYSILPTQVIEDHNSRVESSRLSRDSKWVP
VCPVAISKLYQQGRPILSKVRNLNMQTHSLSRIQVNVDEYAITRRAHFQKMPFFEGPSIPSGGMD
LSELMKSTSLLSLNYDNIKNASLLDMSRVFKCNGSEDDQMAFEFLSDEILEQDVVEEIECNPIF
SISYTKRGESNMTYKNAFHKALISECDKFEEAFDFLDMGFCSNENLSILEEIHWIISYLKTNQWST
ELDNCIHMCMYRNGYDAEYHKFDIPSKFLKDPINRTINWTEVIEFILLIEDFQTKIEPWSSMK
SHFCSKAHSVALECMKNEKRSLAEFVDKSKKTGKSKFDF

Figure S8. Alignment of Gn glycoprotein of OROV (Accession UYI36405) and Schmallenberg virus (SBV) (Accession: YP_009666912.1) shows a percentage identity of 47.41%.

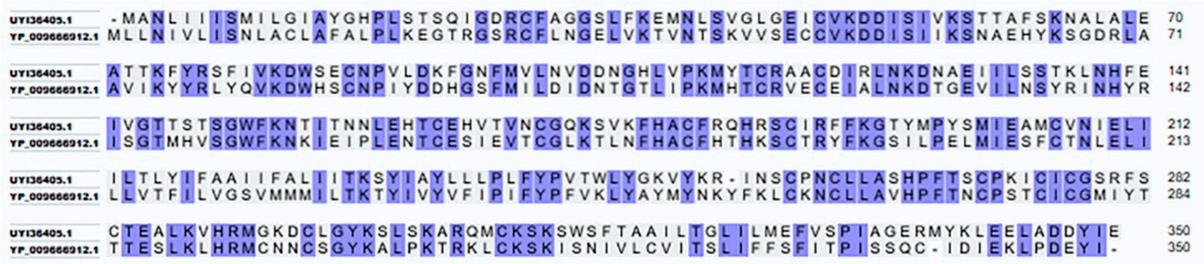

Figure S9. Alignment of Gc glycoprotein of OROV (Accession: UYI36405) and SBV (Accession: YP_009666912.1) shows a percentage identity of 34.59%.

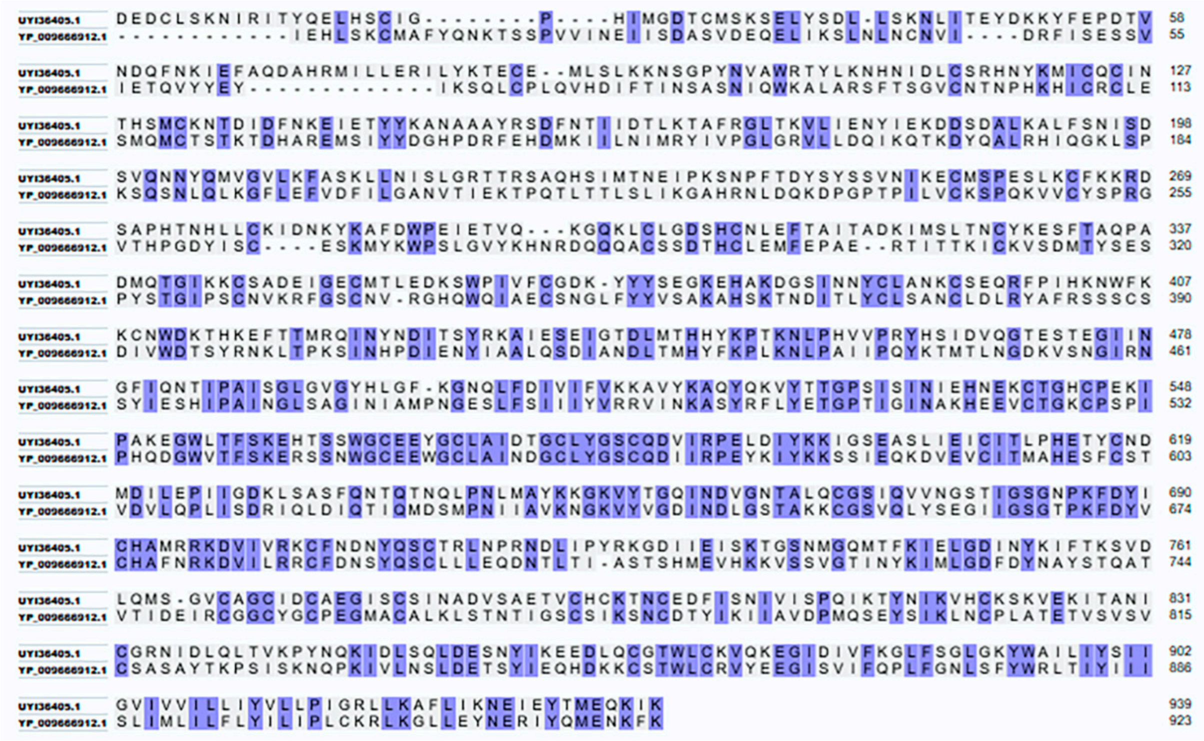

Figure S10. Alignment of NSm of OROV (Accession: UYI36405) and SBV (Accession: YP_009666912.1) shows a percentage identity of 28.95%.

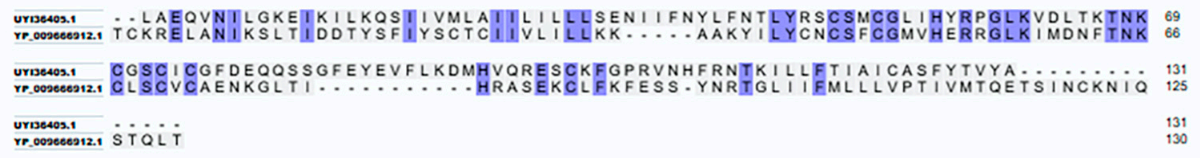

Figure S11. Alignment of NSs of OROV (Accession: AEH03002.1) and SBV (Accession: XCH39114.1) shows a percentage identity of 57.14%.

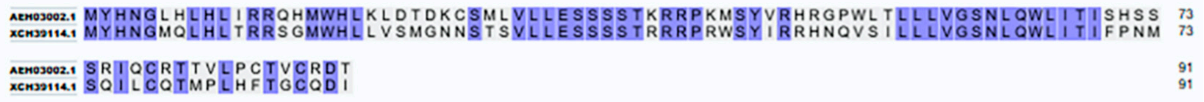

Figure S12. Alignment of nucleocapsid protein of OROV (Accession: AJE24680) and SBV (Accession: XCH39113) shows a percentage identity of 69.70%.

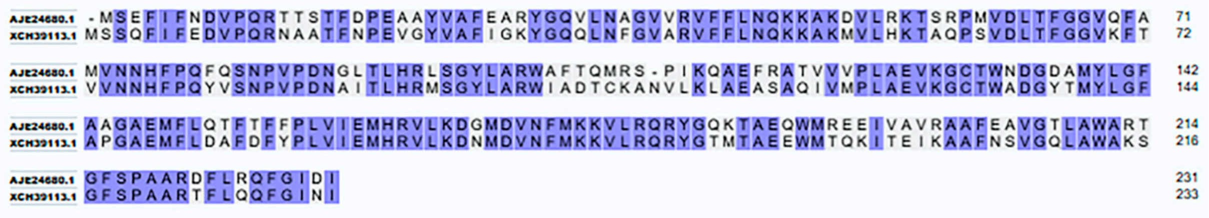

Figure S13. Alignment of RNA polymerase of OROV (Accession: AJE24678) and SBV (Accession: YP_009666911.1) shows a percentage identity of 58.11%.

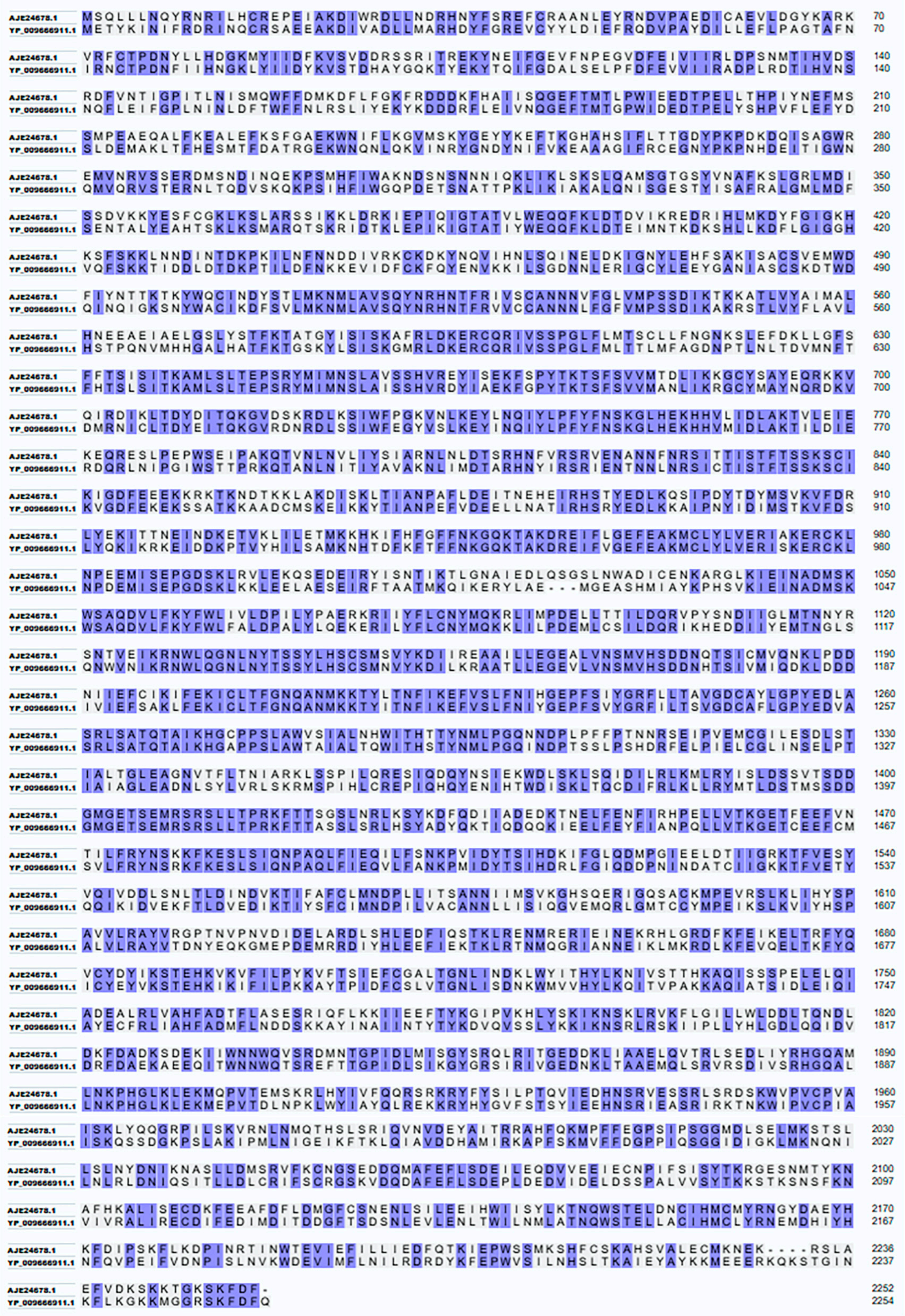

Figure S14. Alignment of Gn glycoprotein of OROV (Accession: UYI36405) and Madre de Dios virus (MDDV) (Accession: AIK67310) shows a percentage identity of 68.86%.

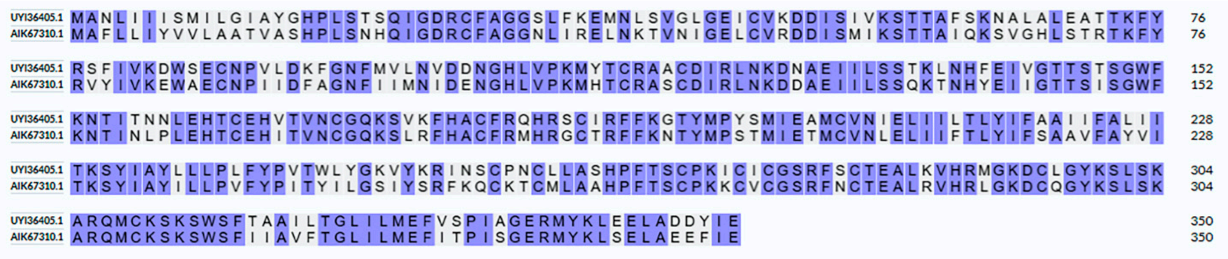

Figure S15. Alignment of Gc glycoprotein of OROV (Accession: UYI36405) and MDDV (Accession: AIK67310) shows a percentage identity of 59.47%.

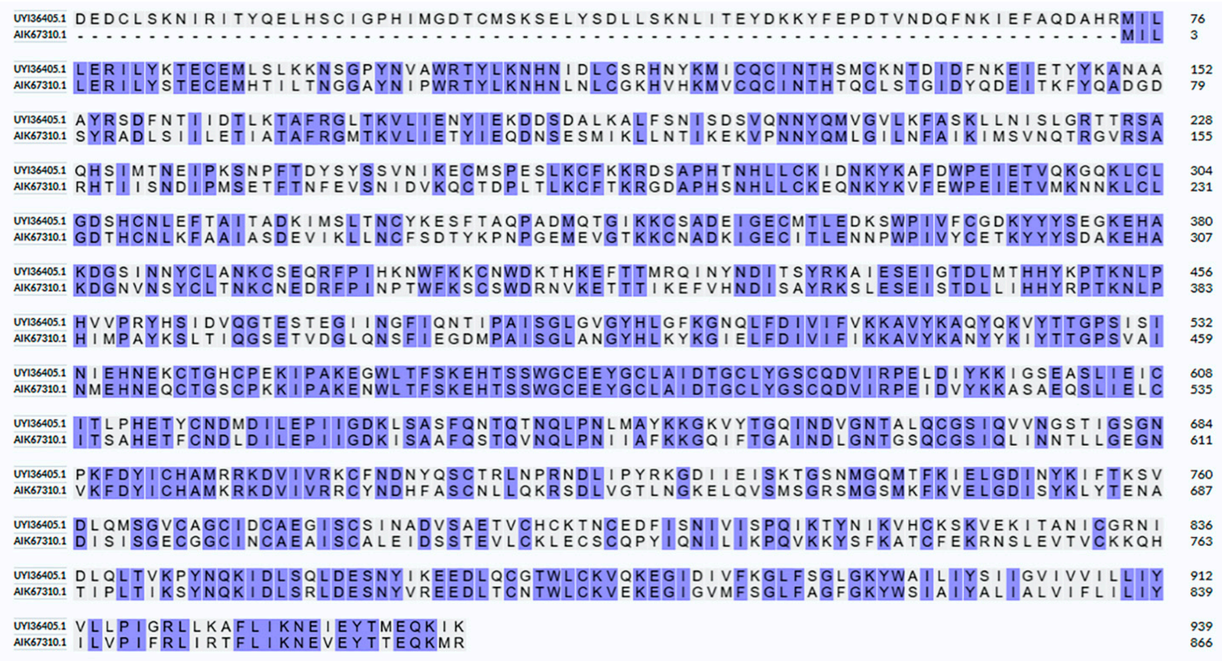

Figure S16. Alignment of non-structural protein NSm of OROV (Accession: UYI36405) and MDDV (Accession: AIK67310) shows a percentage identity of 54.20%.

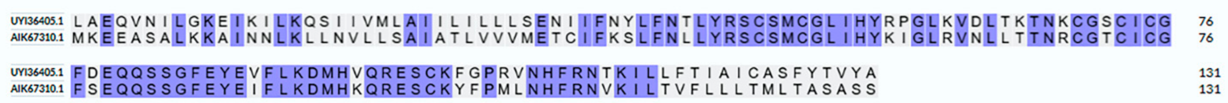

Figure S17. Alignment of non-structural protein NSs of OROV (Accession: AEH03002) and MDDV (Accession: AIK67309) shows a percentage identity of 91.21%.

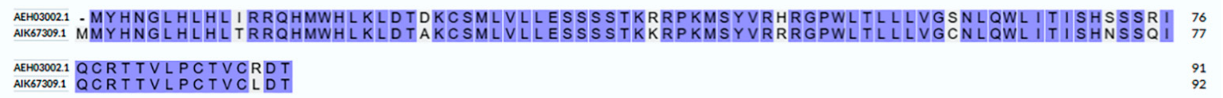

Figure S18. Alignment of nucleocapsid protein of OROV (Accession AJE24680) and MDDV (Accession AHY22351) shows a percentage identity of 100%.

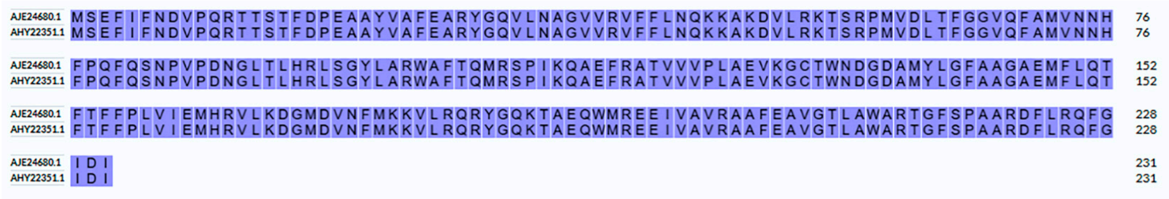

Figure S19. Alignment of RNA polymerase of OROV (Accession AJE24678) and MDDV (Accession AIK67311) shows a percentage identity of 94.89%.

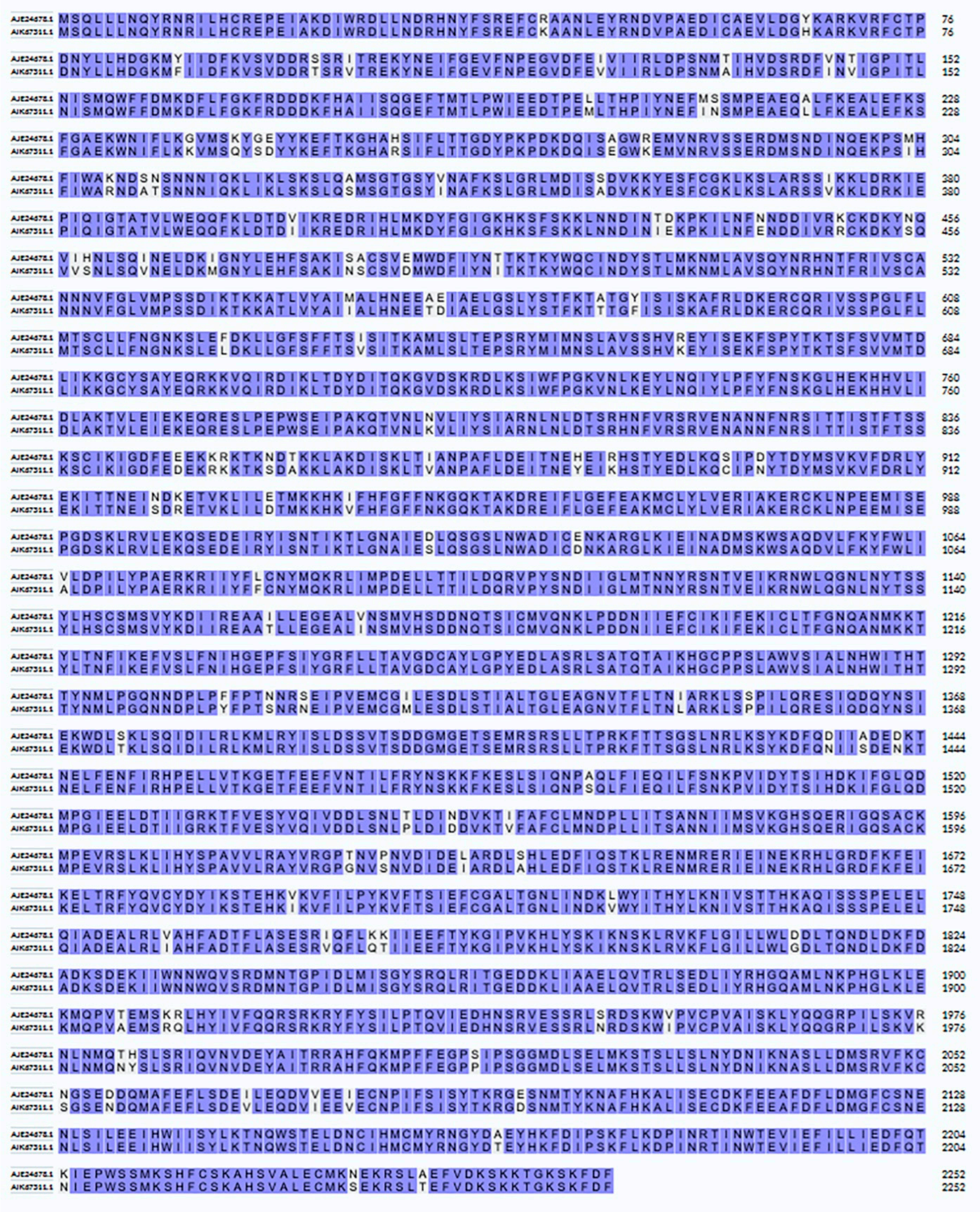

Supplement: Supplementary file 1 [file viruses-17-01434-s001.zip › viruses-3909905-supplementary.pdf]
